# Transverse and oblique course of the vertebral artery over the medullospinal junction

**DOI:** 10.1007/s00276-024-03498-4

**Published:** 2024-10-05

**Authors:** Ana-Maria Davidoiu, Mugurel Constantin Rusu, Corneliu Toader, Petrinel Mugurel Rădoi

**Affiliations:** 1 Doctoral School, Faculty of Medicine, “Victor Babeş” University of Medicine and Pharmacy, Timișoara, RO-300041 Romania; 2 Division of Anatomy, Department 1, Faculty of Dentistry, “Carol Davila” University of Medicine and Pharmacy, RO-020021, Bucharest, 050474 Romania; 3 Division of Neurosurgery, Department 6–Clinical Neurosciences, Faculty of Medicine, “Carol Davila” University of Medicine and Pharmacy, Bucharest, RO-020021 Romania; 4 Clinic of Neurosurgery, “Dr. Bagdasar-Arseni” Emergency Clinical Hospital, Bucharest, RO-041915 Romania

**Keywords:** Foramen magnum, V4 segment of the vertebral artery, Medulla oblongata, Cervical spinal cord, Vertebral artery syndrome

## Abstract

**Purpose:**

The vertebral artery (VA) pierces the dura mater and continues with the intradural V4 segment. Once entered into the dura mater, the VA ascends from the infero-lateral to the antero-superior side of the medulla. Scarce reports of VAs compressing the medullospinal junction (MSJ) are available. We therefore aimed to determine the incidence of a course of the AV over the MSJ.

**Method:**

One hundred sixty-two archived CT angiogram files were documented in the study. We recorded the cases in which the VA crossed the MSJ. We assessed the VA as dominant, non-dominant or co-dominant.

**Results:**

In 32 cases (19.75%), we identified intradural AVs on the ventral side of the MSJs. The incidence of this course of the VA was 17.1% in males and 23.81% in females. Of the 32 cases, the VA was non-dominant in 6, dominant in 14, and co-dominant in 12.

**Conclusion:**

The VA course over the MSJ is not rare. Therefore, when specific neurological signs of MSJ or medulla compression are found, the course of the VA should be documented on CT or MRI angiograms.

## Introduction

The vertebrobasilar system supplies the posterior cerebral circulation. The vertebral artery (VA) ascends in the neck to enter the posterior cranial fossa through the foramen magnum. It further joins the opposite one and forms the basilar artery. The VA has four segments (V1-V4). The V3 segment above the posterior arch of the atlas is extradural, pierces the dura mater, and continues with the intradural segment V4. Once entered into the dura mater, the VA ascends obliquely, anteriorly, superiorly and medially from the infero-lateral side of the medulla to the antero-superior side of it [6, 22].

The VA compression syndrome consists of a broad spectrum of non-specific symptoms due to medullary and/or spinal compression by a VA with a modified course of its V4 segment [14]. Li et al. (2019) state that this VA compression syndrome is unfamiliar to many clinicians and is underrecognised in clinical practice [14]. Scarce reports indicated VAs compressing the medullospinal junction (MSJ) after entering the dura [10, 12, 32]. We, therefore, aimed to determine the incidence of a transverse or transverse/oblique course of the AV over the MSJ in a relevant batch of computed tomography (CT) angiograms.

## Material and method

We used 170 archived CT angiogram files in the study. Inclusion criteria were: good quality of the scans, adequate vertical scans and absence of pathological processes distorting the vascular anatomy. Exclusion criteria were inadequate scans to observe carotid anatomy, perivascular pathological processes distorting the anatomical features, previous surgery in the craniocervical region and posterior fossa, hyperextension or excessive lateral rotation of the neck during CT scan. Eight cases were excluded, and determinations were performed in a retrospective group of 162 cases, 99 males and 63 females. The research followed the principles of the World Medical Association Code of Ethics (Declaration of Helsinki). The Ethics Committee of aff.#4 approved the study (protocol code 2093/01 March 2022).

AngioCT scans were performed with a 32-slice scanner (Siemens Multislice Perspective Scanner, Forcheim, Germany) with 0.6 mm collimation and 0.75 mm thick reconstruction, 50% overlap for maximum intensity multiplanar projection, and a three-dimensional volume rendering technique, as previously described [25]. Cases were documented with the Horos programme for iOS (Horos Project), as in previous studies [18].

We recorded the cases in which the VA was ventral to the MSJ (prejunctional course), crossing or not the midline. We assessed the VA as dominant (D, larger than the contralateral one), non-dominant (ND, thinner than the contralateral one) or co-dominant (CD; the two VAs had comparable calibres). For this, we sequenced axial slices in the caudo-cranial direction, identified the VA penetration into the dural sac, and tracked the ventral relations of the MSJs by correlation with sagittal slices. We verified the results on three-dimensional renderings.

## Results

In 32 cases (19.75%), we identified intradural AVs on the ventral side of the MSJs. Of these 32 cases, 6 had ND VAs (4 left and two right), 14 had D VAs (5 left, nine right), and 12 had CD VAs, five left and seven right.

**Fig. 1:**
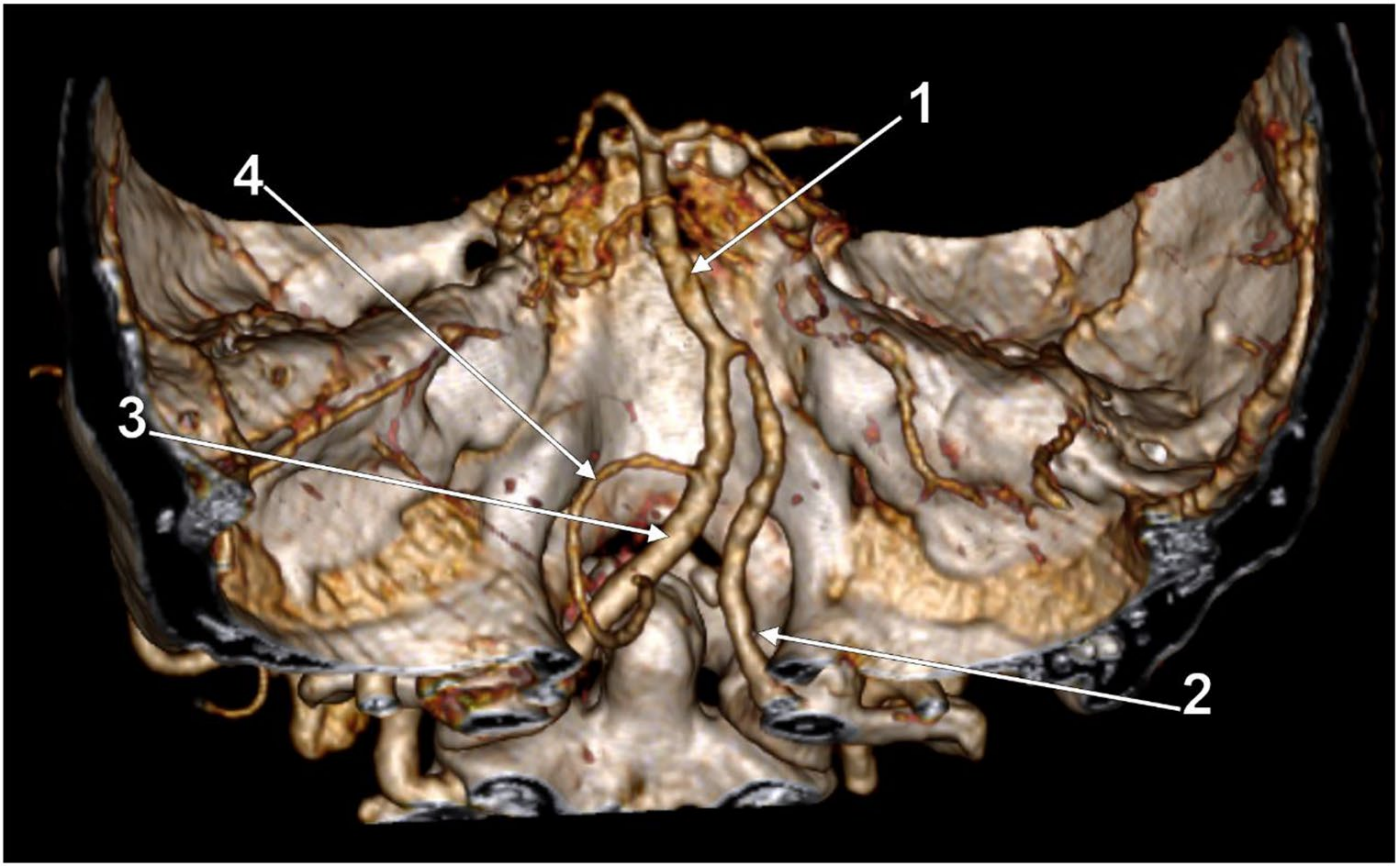
Three-dimensional volume rendering. Posterior view. Left co-dominant vertebral artery with prejunctional course. (**1**) basilar a.; (**2**) right vertebral a.; (**3**) left vertebral a.; (**4**) left posterior inferior cerebellar a

**Fig. 2:**
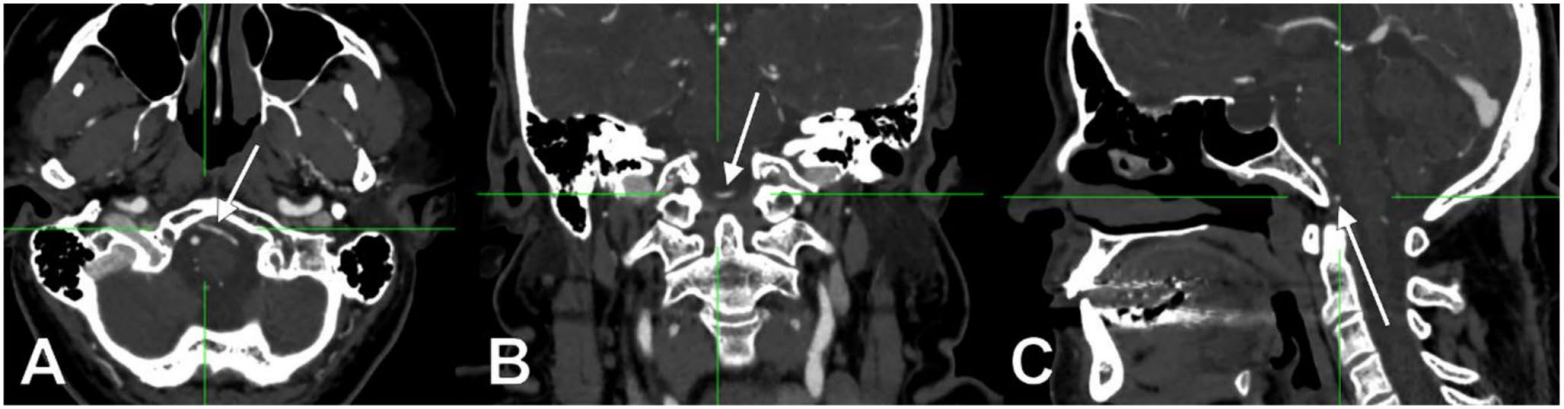
AngioCT slices through the medullospinal junction: axial, inferior view (**A**), coronal, anterior view (**B**) and midsagittal, left lateral view (**C**). Left non-dominant vertebral artery with prejunctional transverse course (arrows)

**Fig. 3:**
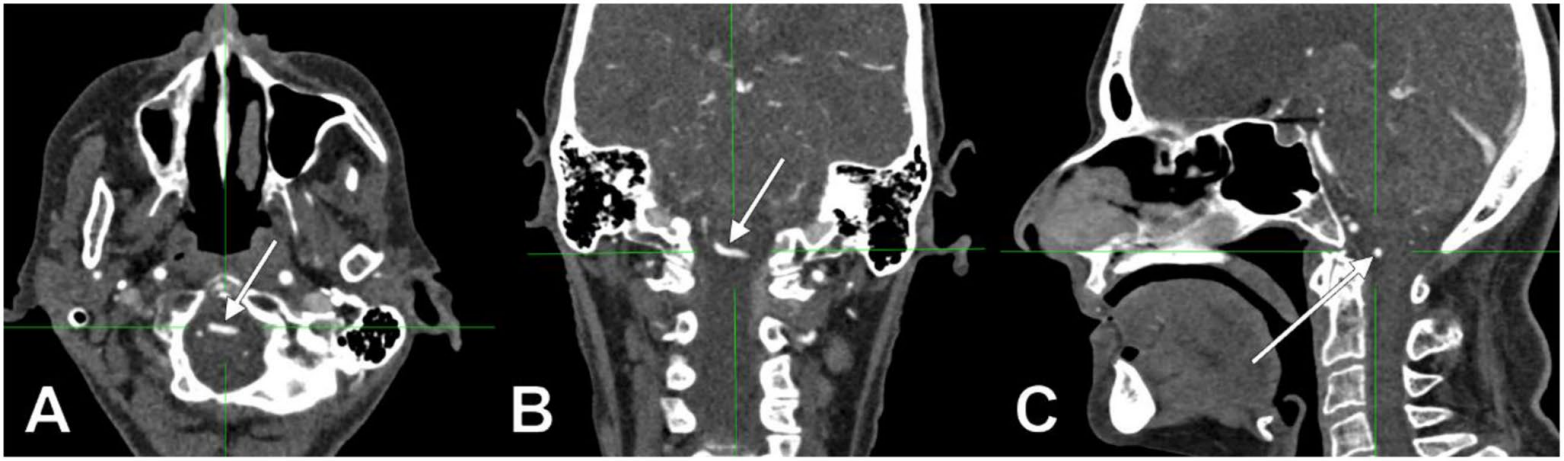
AngioCT slices through the medullospinal junction: axial, inferior view (**A**), coronal, anterior view (**B**) and midsagittal, left lateral view (**C**). Left dominant vertebral artery with prejunctional transverse course (arrows)

In males (N_M_=99), there were 17 positive cases (17.1%). In females (N_F_=63), there were 15 positive cases (23.81%). Of the 17 positive cases in males, five involved left AVs and 12 – right AVs; the AVs were ND in 2/17 cases (11.76%), D in 10/17 cases (58.82%), and CD in 5/17 cases (29.41%, Fig. 1). Of the 15 positive cases in females, nine involved left AVs and 6 – right AVs; the AVs were ND in 4/15 cases (26.67%, Fig. 2), D in 4/15 cases (26.67%, Fig. 3) and CD in 7/15 cases (46.67%).

## Discussion

When the VA has an oblique or even transverse course across the MSJ, it compresses the lateral, but especially the ventral side of the junction. (1) The VA may cause compression of the anterior and lateral corticospinal tracts, which are responsible for fine motor and postural function: depending on the cranial or caudal position to the pyramidal decussation, the symptoms may be ipsi- or contralateral to the area of compression; if the course of the VA is transverse, it may even cause tetraparesis. (2) Compression of the descending vestibulospinal tracts leads to impairment of postural reflexes, with marked disturbances in balance. (3) Compression of the reticulospinal tracts leads to impaired structures responsible for modulating sensory transmission, particularly spinal and pain reflexes. (4) Compression of the tectospinal tract is responsible for the head-turning reflexes; closely related to this is the impairment of the longitudinal medial bundle, which is responsible for the coordination of head and eye movements. (5) On the antero-lateral aspect of the MSJ, the spinothalamic tract may be compressed, causing, in the contralateral territory, thermal, pain and gross tactile sensitivity disorders. (6) In addition, there may be neurological signs of cranial nerve damage: (a) hypoglossal nerve paresis with damage to the extrinsic musculature of the tongue, with hypotrophy of the tongue and deviation of the tip of the tongue, (b) vagus and glossopharyngeal nerve paresis with swallowing and breathing disorders.

Different previous studies [30, 31] did not encounter or report transverse courses of the VA over the MSJ, as in the present study. Tokuda et al. (1985) found 2.3% abnormal atlantoaxial segments of the VA and posterior inferior cerebellar artery [31]. Su et al. (2021) evaluated different dangerous anatomical variants of the VA, such as the persistent first intersegmental artery, fenestration of the VA, dominant VA, and hypoplastic VA [30].

In most existing reports, medullary compressions are determined by atypical courses of the VA [3, 9, 11, 13, 15–17, 19, 21, 24, 27–29]. Such reports were reviewed by Sabet (2021) [26]. Savitz et al. (2006) reported 9 cases, all with the unilateral compression of the anterolateral aspect of the medulla oblongata [28]. Reddy et al. (2023) reported a 3-case series and stated that a medullary compression can be caused by a dolichoectatic VA or a normal dominant VA, or a tortuous, elongated VA [23]. According to these authors, very few cases of medullary compression due to non-dolichoectatic elongated tortuous or dominant AV have been reported in the literature [23]. In 100 cases, there were found 25 unilateral medullary compressions by the VA but none at the MSJ [4], that study being, to our knowledge, the only one establishing an incidence of medullary compression by the VA. A different anatomical pattern in which the VA coursed over the MSJ was the variant in which the artery entered the vertebral canal below the atlas, in the C1/C2 space and continued to the foramen magnum [1, 2, 7, 33]. The upper cervical spinal compression by the VA was also reported [5]. A previously reported case presented a fenestrated VA with an arm in the vertebral canal at the atlantoaxial level; signs of spinal compression were present [8]. In the present study, premature entries of the VA in the vertebral canal were not found.

Cases with specific compression of the MSJ were scarcely reported [10, 12, 20, 32]. Moss and West (1996) found unilateral MSJ compression by a left VA [20]. Koyama (2001) found the bilateral compression of the MSJ by medial loops of the VAs [12]. Ubogu et al. (2002) found the MSJ compression due to a dolichoectatic VA with a medial loop on the ventral side of the junction [32]. Hung and Shen (2011) found the unilateral compression of the MSJ and lateral medulla by a dominant VA [10].

The crossing of the VA and MSJ was found here in 19.75% of cases. Therefore, this anatomic possibility is not rare, and it predisposes to compressions of the neuraxis. Thus, when specific neurological signs of MSJ or medulla compression are found, the course of the VA should be documented on CT or MRI angiograms.

We designed this study as a basic anatomical one, not a clinical one. Leaving from these anatomical findings, further clinical studies could be developed and performed to establish whether an MSJ compression is symptomatic or asymptomatic.

## Data Availability

No datasets were generated or analysed during the current study.
